# Supplementary material for: Antigenic variation of SARS‐CoV‐2 in response to immune pressure
Source: Mol Ecol. 2020 Dec 2;30(14):3548–59. doi: 10.1111/mec.15730 (PMC7753431; doi:10.1111/mec.15730)
Supplement: Supplementary file 4 — Appendix S1 [file MEC-30-3548-s001.docx]

**Text S1. R script for permutation analysis.**

### For each protein we evaluated the difference D between average H values at epitope and non-epitope positions. Most positions of analyzed viruses are invariable along the alignments, so the distribution of H is zero-inflated. We thus calculated statistical significance by permutations. For each protein, the predicted epitope intervals were collapsed to a single position while non-epitope intervals were left unchanged. After randomly shuffling this collapsed sequence it was expanded back to full length and the difference between shuffled epitope and non-epitope H values was calculated. This procedure was repeated 1000 times and the proportion of repetitions showing a difference more extreme than D was reported as p-value.

permuteEpitopes<-function(epitopeAnnotation,entropy,N=1000){

collapseEpitope<-function(epi){

posprotein<-1;

poslista<-0;

L<-list();

prev<-0

while(posprotein<=length(epi)){

if(is.na(epi[posprotein])){

poslista<-poslista+1;

L[[poslista]]<-NA;

}else if(epi[posprotein]==0 & !is.na(epi[posprotein])){

poslista<-poslista+1;

L[[poslista]]<-0;

}else if (epi[posprotein]==1 & !is.na(epi[posprotein])){

if(prev==0 | is.na(prev) ){

poslista<-poslista+1

}

if(poslista>length(L)){

L[[poslista]]<-1;

}else{

L[[poslista]]<-c(L[[poslista]],1);

}

}

prev<-epi[posprotein]

posprotein<-posprotein+1;

}

L

}

shuffleAndExpandEpitope<-function(L,s=TRUE){

ids<-1:length(L)

if(s){

ids<-sample(ids)

}

epi<-c()

for(i in 1:length(ids)){

epi<-c(epi,L[[ids[i]]])

}

epi

}

d1<-rep(NA,N)

d0<-rep(NA,N)

L<-collapseEpitope(epitopeAnnotation)

for(i in 1:N){

sepi<-shuffleAndExpandEpitope(L)

d1[i]<-mean(entropy[which(sepi==1)],na.rm=T)

d0[i]<-mean(entropy[which(sepi==0)],na.rm=T)

}

D<-mean(entropy[which(epitopeAnnotation==1)],na.rm=T)-mean(entropy[which(epitopeAnnotation==0)],na.rm=T)

if(D>=0){

ret<-list(Delta=D,p.value=mean(d1-d0>=D))

}else{

ret<-list(Delta=D,p.value=mean(d1-d0<=D))

}

ret

}
